# Growth on Formic Acid Is Dependent on Intracellular pH Homeostasis for the Thermoacidophilic Methanotroph *Methylacidiphilum* sp. RTK17.1

**DOI:** 10.3389/fmicb.2021.651744

**Published:** 2021-03-24

**Authors:** Carlo R. Carere, Kiel Hards, Kathryn Wigley, Luke Carman, Karen M. Houghton, Gregory M. Cook, Matthew B. Stott

**Affiliations:** ^1^Department of Chemical and Process Engineering, University of Canterbury, Christchurch, New Zealand; ^2^Department of Microbiology and Immunology, University of Otago, Dunedin, New Zealand; ^3^Maurice Wilkins Center for Molecular Biodiscovery, Auckland, New Zealand; ^4^Geomicrobiology Research Group, Department of Geothermal Sciences, GNS Science, Taupō, New Zealand; ^5^School of Biological Sciences, University of Canterbury, Christchurch, New Zealand

**Keywords:** methanotroph, acidophile, pH homeostasis, *Methylacidiphilum*, formate, formic acid

## Abstract

Members of the genus *Methylacidiphilum*, a clade of metabolically flexible thermoacidophilic methanotrophs from the phylum Verrucomicrobia, can utilize a variety of substrates including methane, methanol, and hydrogen for growth. However, despite sequentially oxidizing methane to carbon dioxide *via* methanol and formate intermediates, growth on formate as the only source of reducing equivalents (i.e., NADH) has not yet been demonstrated. In many acidophiles, the inability to grow on organic acids has presumed that diffusion of the protonated form (e.g., formic acid) into the cell is accompanied by deprotonation prompting cytosolic acidification, which leads to the denaturation of vital proteins and the collapse of the proton motive force. In this work, we used a combination of biochemical, physiological, chemostat, and transcriptomic approaches to demonstrate that *Methylacidiphilum* sp. RTK17.1 can utilize formate as a substrate when cells are able to maintain pH homeostasis. Our findings show that *Methylacidiphilum* sp. RTK17.1 grows optimally with a circumneutral intracellular pH (pH 6.52 ± 0.04) across an extracellular range of pH 1.5–3.0. In batch experiments, formic acid addition resulted in no observable cell growth and cell death due to acidification of the cytosol. Nevertheless, stable growth on formic acid as the only source of energy was demonstrated in continuous chemostat cultures (D = 0.0052 h^−1^, t_d_ = 133 h). During growth on formic acid, biomass yields remained nearly identical to methanol-grown chemostat cultures when normalized per mole electron equivalent. Transcriptome analysis revealed the key genes associated with stress response: methane, methanol, and formate metabolism were differentially expressed in response to growth on formic acid. Collectively, these results show formic acid represents a utilizable source of energy/carbon to the acidophilic methanotrophs within geothermal environments. Findings expand the known metabolic flexibility of verrucomicrobial methanotrophs to include organic acids and provide insight into potential survival strategies used by these species during methane starvation.

## Introduction

The aerobic methane-oxidizing bacteria (methanotrophs) are able to grow exclusively on methane (CH_4_) as their sole source of carbon and energy (Whittenbury et al., 1970). They provide vital ecosystem function by serving as the primary biological sink for methane (Chistoserdova, 2015; Knief, 2015) and are of biotechnological interest in the development of commercial gas-to-liquid (Kalyuzhnaya et al., 2015) and proteinaceous feedstock (Strong et al., 2016) bioprocesses. Methanotrophs oxidize methane to methanol *via* a particulate or soluble methane monooxygenase enzyme (pMMO/sMMO) and subsequently yield reducing equivalents (e.g., NADH) for cellular respiration and biosynthesis through the oxidation of methanol to carbon dioxide. The gammaproteobacterial (Type I) and alphaproteobacterial (Type II) methanotrophs assimilate the intermediate formaldehyde using the ribulose monophosphate (RuMp) or serine pathways (Hanson and Hanson, 1996), respectively. In contrast, methanotrophs within the phylum Verrucomicrobia (genera *Methylacidiphilum* and *Methylacidimicrobium*) oxidize methanol directly to formate (Keltjens et al., 2014) and grow autotrophically by fixing carbon dioxide (CO_2_) *via* the Calvin-Benson-Bassham cycle (Khadem et al., 2012c). For all known methanotrophs, the enzyme formate dehydrogenase (FDH) catalyzes the terminal step of methane oxidation, yielding NADH and CO_2_. There is considerable heterogeneity in the structure and composition of bacterial formate dehydrogenases with multiple copies of FDH-encoding genes commonly found in the genomes of methanotrophic and methylotrophic bacteria (Ferry, 1990; Yoch et al., 1990; Ward et al., 2004).

While the majority of studies have emphasized that methanotrophic bacteria have limited metabolic flexibility (Whittenbury et al., 1970; Smith and Hoare, 1977; Shishkina and Trotsenko, 1982; Wood et al., 2004; Kelly et al., 2005), several investigations have now overturned the paradigm of “obligate methanotrophy” (Dedysh and Dunfield, 2010). A few strains, notably *Methylocella silvestris* and other *Methylocella* species, utilize simple organic acids, alcohols, and short-chain alkane gases for growth (Dedysh et al., 2005; Crombie and Murrell, 2014). Aerobic H_2_ metabolism has also been reported in several methanotrophs (Chen and Yoch, 1987; Shah et al., 1995; Hanczar et al., 2002; Hakobyan et al., 2020), and hydrogenase-encoding genes are ubiquitously distributed in methanotroph genomes (Greening et al., 2016). In the acidophilic verrucomicrobial methanotrophs, chemolithoautotrophic growth on H_2_ has been reported (Mohammadi et al., 2016, 2019; Carere et al., 2017), with mixotrophic growth (H_2_ and CH_4_) proposed to provide a competitive advantage over obligate methanotrophy at the oxic/anoxic soil boundaries of acidic geothermally heated soils (Carere et al., 2017). A recent report that *Methylacidiphilum fumariolicum* SolV can utilize ethane and propane gases has further expanded the suit of substrates that can support the survival of these methanotrophs *in situ* (Picone et al., 2020).

Methanotrophs thrive at the interface of many oxic/anoxic habitats (e.g., peat bogs, forest soils, wetlands, rice paddies, geothermal, and volcanic environments; Dunfield et al., 2007; Singh et al., 2010; Knief, 2015) where the availability of oxidant (O_2_), energy, and carbon resources for growth fluctuate (Knief et al., 2003; Tavormina et al., 2010). In these habitats, organic polymers (i.e., lignocellulose) may anaerobically decompose into organic acids, alcohols, molecular hydrogen (H_2_), and carbon dioxide (CO_2_) and subsequently diffuse into oxic environments to facilitate aerobic growth. In general, low molecular weight organic acids display low concentrations and a short residence time in soils (Angeles et al., 2006); however, the accumulation of formic (0.65 mM), acetic (0.26 mM), and lactic acids (0.085 mM) in acidic wetland soil porewaters (Küsel et al., 2008) is illustrative that these compounds represent available substrates for microbial growth *in situ*.

Organic acids, such as formic acid, however, are potentially harmful to acidophilic species because they can function as uncouplers of the respiratory chain. In acidic conditions (pH < 3), diffusion of the protonated form (i.e., CHOOH; pK_a_ 3.74) into the cell is followed by rapid proton dissociation and cytosolic acidification (i.e., CHOO^−^ + H^+^) that ultimately can collapse the proton motive force governing respiratory-linked ATP synthesis (Alexander et al., 1987; Kishimoto et al., 1990; Ciaramella et al., 2005; Baker-Austin and Dopson, 2007; Lund et al., 2014). Accordingly, the genomes of many acidophilic species encode for enzymes to actively degrade organic acids. Thus, although cells may gain energy from their oxidation, the genomic constituents of organic acid degradation may primarily serve a role in pH homeostasis. Despite the ubiquity of FDH in sequenced acidophilic methanotroph genomes (*Methylacidiphilum* spp., *Methylacidimicrobium* spp.), no studies have demonstrated the metabolic capability to grow using formate or other organic acids as a sole energy source (Op den Camp et al., 2009; van Teeseling et al., 2014).

In this work, we investigated growth and intracellular pH homeostasis in response to formate/formic acid addition within the thermoacidophilic methanotroph *Methylacidiphilum* sp. RTK17.1. Demonstrating that *Methylacidiphilum* sp. RTK17.1 can grow organoautotrophically on formic acid expands the known spectrum of substrates used by verrucomicrobial methanotrophs to include organic acids and provides important new insights into the physiology and ecology of these acidophiles.

## Materials and Methods

### Cultivation of *Methylacidiphilum* sp. RTK17.1

*Methylacidiphilum* sp. RTK17.1 was previously isolated from geothermally heated soils sampled from Rotokawa, New Zealand (Carere et al., 2017), and shares 99% 16S rRNA gene sequence identity to *Methylacidiphilum infernorum* V4. All batch cultivations were performed at 50°C in a V4 mineral medium as described previously (Dunfield et al., 2007) but with the addition (0.2 μM) of rare earth elements lanthanum and cerium (Pol et al., 2014) unless otherwise specified. To determine the pH range for the growth of *Methylacidiphilum* sp. RTK17.1, 4 ml of pH-adjusted V4 media (range: pH 0.5–6.0; H_2_SO_4_) was made, added to (20 ml) Balch tubes (Bellco), and sealed with butyl rubber stoppers prior to sterilization. After autoclaving (121°C, 15 psi, 20 min), air headspaces were supplemented with methane (~10% v/v) and CO_2_ (~5% v/v). Tubes were inoculated with exponentially growing *Methylacidiphilum* sp. RTK17.1 cells to an initial OD_600nm_ of 0.02. The maximum specific growth rates (μ_max_) were determined from the exponential phase of cultures spectrophotometrically (600 nm). The concentration of CH_4_ in the headspace was monitored with a PeakPerformer gas chromatograph (Peak Laboratories) outfitted with a HayeSepD column and equipped with a flame ionizing detector (FID) by injecting 1 ml samples *via* a gas-tight syringe (SGE Analytical Science, Melbourne).

Chemostat cultivation was performed to investigate the growth of *Methylacidiphilum* sp. RTK17.1 on methanol and formic acid. For these experiments, cells were cultivated in a bioreactor with a working volume of 2 L (LABFORS 3; max volume 3.6 L) at pH 2.5, 45°C, and with continuous agitation at 450 rpm. A gas headspace composition of 2% O_2_ and 2% CO_2_ (v/v; balance N_2_) was supplied continuously at 150 ml min^−1^ *via* mass flow control throughout the duration of these experiments. Initially, cells were grown on methanol (12.4 mM) in a fed-batch mode, such that exhaustion of methanol (as determined spectrophotometrically *via* cessation of cell growth) prompted repeated dosing of additional methanol back to a concentration of 12.4 mM. Once cell density reached OD_600nm_ ~0.5, the bioreactor was switched to chemostat operation for continuous growth experiments. During chemostat growth experiments, V4 mineral medium was supplemented with either 12.4 mM methanol or 12.4 mM formic acid and supplied at a constant flow rate of 10.4 ml h^−1^ (D = 0.0052 h^−1^, t_d_ = 133 h). After achieving a steady state, cultures were monitored over a period of several days in order to determine biomass productivity data. During this time, biomass samples of *Methylacidiphilum* sp. RTK17.1 were harvested directly from the bioreactor for transcriptome sequencing and high-performance liquid chromatography (HPLC) analysis. Steady state methanol and formic acid concentrations (from feed medium and bioreactor samples) were quantified using a Thermo UltiMate 3000 HPLC (Dionex) equipped with an Aminex HPX-87H column (300 × 7.8 mm) and refractive index and UV vis (210 nm) detectors. A 5 mM H_2_SO_4_ mobile phase was provided at a constant flowrate of 0.5 ml min^−1^. Resultant chromatogram profiles were integrated using tools embedded within the Chromeleon 7.2 SR5 software. Effluent biomass (500 ml) was collected over this period for cell dry weight determinations. For this analysis, cell pellets were collected by centrifugation (10,000 rpm, 10 min, 25°C; Eppendorf Centrifuge 5810 R) and then dried to constant mass using a Labconco FreeZone 2.5 freeze dryer.

### Bioenergetic Determinations

Cell suspensions of *Methylacidiphilum* sp. RTK17.1 were prepared in V4 medium (pH 2.5) from methane-grown cultures to a final OD_600nm_ 0.8–1.0 as previously described (Carere et al., 2017). Previously, the energization of cell suspensions *via* the catabolism of intracellular glycogen reservoirs was confirmed by O_2_ consumption assays using a Digital model 10 Oxygen electrode (Rank Brother ltd., United Kingdom) in the presence of cyanide *m*-chlorophenyl hydrazone (CCCP), the glycolytic inhibitor iodoacetamide (IAA), and the respiratory chain inhibitor potassium cyanide (KCN; Carere et al., 2017).

Cytosolic pH, membrane potential (Δψ), and intracellular volume determinations were performed as described previously (Cook et al., 1996). Briefly, the intracellular volume (3.45 ± 0.59 μl mg protein^−1^) was estimated from the difference between the partitioning of ^3^H_2_O and [7-^14^C]benzoic acid. The Δψ was calculated from the uptake of the lipophilic cation [^14^C]tetraphenylphosphonium (TPP^+^) according to the Nernst relationship. Non-specific isotope binding was estimated from cells, which had been treated with valinomycin (10 μM) and/or nigericin (10 μM) for 25 min. The ∆pH was determined from the distribution of [carboxyl-^14^C]salicylic acid (56 mCi mmol^−1^) using the Henderson-Hasselbalch equation (Riebeling et al., 1975), and Z∆pH was calculated as 62 mV × ∆pH. Following incubation for 20 min at 50°C, *Methylacidiphilum* sp. RTK17.1 cell suspensions were centrifuged through a silicon oil mixture (equal parts Dexter Hysol 550 and 560; Hysol Co., Oleam, NY) in microcentrifuge tubes (13,000 × *g*, 5 min), and 20-μl samples of supernatants were removed. The tubes and contents were then frozen (−20°C), and the bottoms (containing cell pellets) were subsequently removed with dog nail clippers. Supernatants and cell pellets were dissolved in scintillation fluid (Optiphase Hisafe 2; Scitech Biolab) and counted.

Next, the influence of formic acid dosing on intracellular pH homeostasis was determined. To perform these experiments, formic acid was added to non-growing, but energized, cultures of *Methylacidiphilum* sp. RTK17.1 (methane-grown) at concentrations ranging from 0.01 to 1,000 mM. Cells were pelleted by centrifugation, suspended in V4 medium (OD_600nm_ ~1.0) supplemented with formic acid (pH adjusted to 2.5 with 1 M H_2_SO_4_), and incubated for 20 min at 50°C prior to intracellular pH determinations (as described above).

### Formate Dehydrogenase Assays

Biomass (1 l) collected from *Methylacidiphilum* sp. RTK17.1 cells grown in batch on methane (Carere et al., 2017) was used to determine the activity of formate dehydrogenase at different pH values. Briefly, 1 l of cells (OD_600nm_ 0.810) was pelleted by centrifugation at 4,500 rpm for 20 min at 4°C and stored at −80°C until required for further processing. The resultant cell pellet was then suspended in a lysis buffer (10 ml) containing 50 mM Tris-HCl (pH 8.0), 10 μM methyl viologen, and 25 mM β-mercaptoethanol and then disrupted by sonication at 15 μF (10 × 30 s) on ice. Cell lysis (>99%) was confirmed microscopically, after which the crude lysate was centrifuged at 4,000 rpm for 30 min (4°C) to collect cell debris. The resultant cell-free extracts (2 ml) were then anaerobically transferred into N_2_-flushed stoppered glass assay tubes containing 2 ml of lysis buffer supplemented with 10 mM formate, gassed/degassed three times with N_2_ (3 min/3 min), and preincubated at 50°C for 10 min. An assay solution was prepared containing 100 mM select phosphate buffer (pH 5.5–8.0), 2 mM methyl viologen, 20 mM formate, and 10 mM β-mercaptoethanol and then gassed/degassed three times with N_2_ (3 min/3 min). Enzymatic reactions were then initiated by the addition of 100 μl of preincubated cell-free extracts into assay solution-containing tubes, and the time-dependent reduction of methyl viologen was recorded spectrophotometrically (600 nm). All assays were performed in triplicate, and the activity of formate dehydrogenase was presented as a percentage of the rate observed at pH 7.

### Transcriptome Sequencing

Samples for transcriptome sequencing were harvested (10 ml) from exponential (methanol) and steady-state (methanol, formic acid) phases of chemostat experiments. *Methylacidiphilum* sp. RTK17.1 cells were pelleted by centrifugation at 5,000 × *g* (15 min, 4°C), suspended in 300 μl of RNAlater Stabilization solution (ThermoFisher Scientific) and then stored at −20°C until required for further analysis (as per the manufacturer’s recommended protocols). Total RNA was extracted using the Mo Bio PowerBiofilm RNA Isolation kit following the manufacturers recommended protocol, eluted into 100 μl of ddH_2_O, and quantified using the Qubit HS assay kit (Thermo Fisher Scientific). Following this, 2 μg of RNA was transferred into RNAstable preservation medium (Sigma Aldrich) and dehydrated at room temperature before transport for transcriptome sequencing (Custom Science; China). Upon receipt of samples, RNA integrity was confirmed using an Agilent 2100 Bioanalyser.

Following total RNA extraction, ribosomal RNAs were removed using the Ribo-Zero rRNA removal kit (bacteria), and the quality of the remaining RNA was assessed using an Agilent 2100 Bioanalyzer (Agilent). Library construction was performed using the TruSeq Stranded Total RNA Sample Prep (microbe) Kit (Illumina), and sequencing was performed using an Illumina HiSeq2500 platform. From this, an average of 10,452,057 raw untrimmed reads were obtained for each of the conditions sampled (methanol exponential, methanol steady state, and formic acid steady state). These reads were then analyzed using the Artificial Intelligence RNA-Seq pipeline (Sequentia Biotech, Barcelona, Spain; Vara et al., 2019), which were reduced to an average of 9,154,571 reads following quality filtering and trimming. Retained paired-end reads (100 bp) were then mapped to the genome of *Methylacidiphilum infernorum* strain V4 (GCA_000019665.1; Hou et al., 2008).

Differential gene expression profiles and accompanying statistical analysis was performed to investigate transcriptional regulation using edgeR (Robinson et al., 2009). Synonymous conditions (methanol exponential, methanol steady state) were grouped as replicates for differential gene expression analysis and compared with the formic acid growth condition. Where indicated, comparisons to methane-grown *Methylacidiphilum* sp. RTK17.1 cultures refer to transcriptomes described previously (accession numbers GSM3872525-GSM3872529; Carere et al., 2019). Using these additional transcriptomes, samples were partitioned into five groups [formic acid (*n* = 1), methanol (*n* = 2), CH_4_ with O_2_ limitation (*n* = 2), CH_4_ with O_2_ limitation and N_2_-fixing (*n* = 2), and CH_4_ with excess O_2_ (*n* = 2)] to estimate the biological coefficient of variation within the dataset. Using tools within edgeR, multi-dimensional scaling and sample distance matrix analysis then was performed to verify that formic acid, methanol, and methane growth conditions were sufficiently different from one another. Expression values are provided as either fragments per kilobase million (FPKM; Supplementary Table S1) values (Mortazavi et al., 2008) or Z-normalized FPKM (Hart et al., 2013). Raw and processed transcriptome sequence files (accession number GSE145277) are available in the Gene Expression Omnibus (GEO) under accession numbers GSM4314091–GSM4314093.

## Results and Discussion

### *Methylacidiphilum* sp. RTK17.1 Maintains a Circumneutral Cytosolic pH That Is Sensitive to Organic Acid-Induced pH Stress

**Figure 1:**
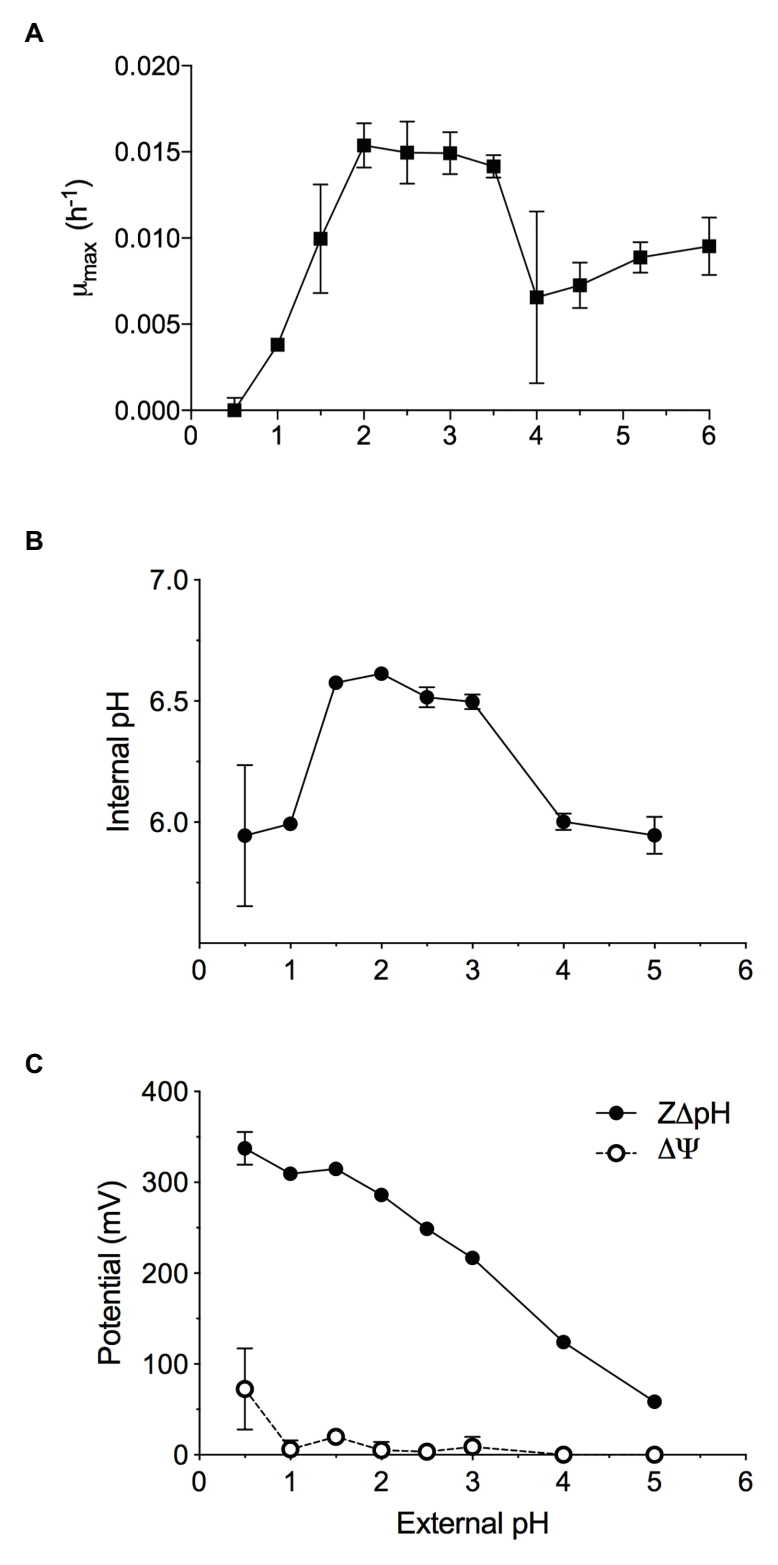
The maximal growth rate of methane-grown *Methylacidiphilum* sp. RTK17.1 in batch culture is highly dependent on maintaining pH homeostasis. Optimal growth was observed where extracellular 1.5 < pH < 4.0, resulting in the maintenance of an internal pH 6.55 ± 0.05. **(A)** Maximum specific growth rates observed in batch cultures of *Methylacidiphilum* sp. RTK17.1 (10% CH_4_ v/v in air) at 50°C in V4 medium at different pHs (range pH 0.5–6.0). **(B)** The corresponding internal pH and; **(C)** membrane potential (Δψ) and (Z∆pH) as a function of external pH are shown. Error bars represent the standard deviation of triplicate measurements.

*Methylacidiphilum* sp. RTK17.1 was observed to grow methanotrophically between pH 1.0 and 6.0 (pH_opt_: ~2.5; μ_max_: 0.015 h^−1^; Figure 1A) in batch culture experiments. These results approximate reports of growth in the other known verrucomicrobial thermoacidophilic methanotrophs: *Methylacidiphilum infernorum* V4 (Dunfield et al., 2007), *Methylacidiphilum kamchatkensis* Kam1 (Islam et al., 2008), *Methylacidiphilum fumariolicum* SolV (Pol et al., 2007). Although growth down to pH 0.8 has been reported in *Methylacidiphilum fumariolicum* SolV, growth at values of pH < 1.0 was not observed in cultures of *Methylacidiphilum* sp. RTK17.1 and appears to be a trait more common with the mesophilic strains of the Methylacidiphilales (van Teeseling et al., 2014).

**Figure 2:**
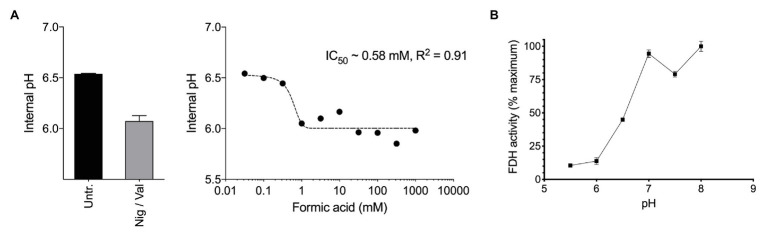
**(A)** Exposure of methane-grown batch cultures of *Methylacidiphilum* sp. RTK17.1 to increasing concentrations of formic acid (pk_a_ = 4.3) leads to cytosol acidification. A nonlinear fit was used to estimate the half maximal inhibitory concentration of formic acid (IC_50_ = 0.58 mM, R^2^ = 0.91). The internal pH of untreated (Untr.) cell suspensions and samples treated with 10 μM each nigericin and valinomycin (Nig/Val) are shown. **(B)** Formate dehydrogenase (FDH) activity, as determined from crude cell extracts of methane-grown *Methylacidiphilum* sp. RTK17.1 cultures is maximal at the circumneutral pH values characteristic of cytosolic pH optima. Results are presented as a percentage of the rate at pH 7. Error bars represent the standard deviation of triplicate measurements.

Similar to neutralophilic bacteria, acidophiles must maintain circumneutral intracellular pH in order to remain viable (Baker-Austin and Dopson, 2007). However, very little is understood about how acidophilic species manage the considerable pH gradients that exist between the extracellular environment and the cytosol. We sought to investigate pH homeostasis in *Methylacidiphilum* sp. RTK17.1 in response to external acid stress. When non-growing, but energized, *Methylacidiphilum* sp. RTK17.1 cells at pH 2.5 display an intracellular pH of 6.52 ± 0.04 (Figure 1B). Likewise, similar intracellular pH values (6.55 ± 0.05) were obtained at extracellular values between pH 1.5 and 3.0. These circumneutral values approximate the known cytoplasmic pH for other acidophilic bacteria, including *Acidithiobacillus ferrooxidans* (pH 6.5), *Acidithiobacillus thiooxidans* (pH ~7), and *Acidiphilium acidophilum* (pH 6.0; Baker-Austin and Dopson, 2007) but differ markedly from the archaeon *Picrophilus oshimae*, which maintains an intracellular value of pH 4.6 when extracellular pH is <4.0 (van de Vossenberg et al., 1998). When *Methylacidiphilum* sp. RTK17.1 was incubated at pH values analogous to a decreased observed rate of growth (1.0 > pH > 3.0), cytosolic acidification (pH 5.97 ± 0.13) was observed. A similar degree of cytosolic acidification was observed in cell suspensions following treatment with nigericin/valinomycin (10 μM each; Figure 2A). The lack of complete pH gradient dissipation is consistent with previous reports of uncoupler treatment and has been attributed to both the limited cation membrane permeability and high cytoplasmic buffering capacity characteristic of acidophiles (Goulbourne et al., 1986; Baker-Austin and Dopson, 2007).

The chemical gradient of protons (∆pH) across the cell membrane is a major contributor to the proton motive force (PMF; PMF = Δψ − Z∆pH) driving ATP production *via* the electron transport chain. This large ∆pH is actively maintained in acidophiles and is further supported by a “reversed” membrane potential (Δψ) that is cytosol (inside)-positive relative to the extracellular (outside) environment. This is opposite to the cytosol-negative Δψ of neutralophiles. Indeed, at extracellular pH values between pH 2 and 3, *Methylacidiphilum* sp. RTK17.1 maintains a minimal membrane potential (Δψ = 5.86 mV, Figure 1C) that is “reversed.” These data are comparable to values reported for other known acidophiles, including *A. ferrooxidans* and *Alicyclobacillus acidocaldarius* (Krulwich et al., 2011), and *Thiobacillus acidophilus* (Zychlinsky and Matin, 1983). The inside positive Δψ contributes to inhibiting the entry of extracellular protons and detracts from the large proton motive force formed by this ΔpH.

While the mechanisms used by acidophiles to maintain pH homeostasis permit respiration and growth in acidic environments (Baker-Austin and Dopson, 2007), the ∆pH has a detrimental effect of increasing susceptibility of these bacteria to organic acid-induced pH stress. This is because the conjugate acid-base pairing of organic compounds (e.g., formate/formic acid) strongly favors the protonated form in acidic environments. When organic acids diffuse from an acidic extracellular environment across the cell membrane and into the cytosol (circumneutral pH), the conjugate-base anion dissociates, releasing protons (Baker-Austin and Dopson, 2007; Krulwich et al., 2011). At elevated organic acid concentrations, these liberated protons can act as respiratory chain uncouplers by collapsing the ∆pH. For the acidophilic methanotrophs occupying oxic/anoxic interfaces in acidic geothermal environments, this scenario poses a problem, as these species are likely to encounter organic acids. It is unknown how acidophilic methanotrophs cope with organic acid-induced pH stress, but given that formate oxidation is the terminal step common to all aerobic methanotrophs, they should be poised to gain energy from environmental fluxes of formic acid.

Therefore, we next investigated how formic acid stress affected cytosolic pH and formate dehydrogenase (FDH) activity in *Methylacidiphilum* sp. RTK17.1. In a dose-dependent manner, we observed that the addition of formic acid to non-growing cultures of *Methylacidiphilum* sp. RTK17.1 resulted in cytosolic acidification (Figure 2A), which resembled external pH stress and uncoupler-treatment experiments. Intracellular pH decreased from pH 6.52 to 6.05 with the addition of 1 mM formic acid. We interpret the absence of further cytosolic acidification with increasing formic acid concentrations (up to 1 M) as a consequence of cytoplasmic buffering that has previously been reported (Zychlinsky and Matin, 1983; Goulbourne et al., 1986; Baker-Austin and Dopson, 2007).

Formate dehydrogenase activities from crude cell extracts using the artificial electron acceptor methy-viologen were maximal between pH 7.0 and 8.0; consistent with the internal pH optima (Figure 2B). FDH activities, however, decreased to 13.8% of the maximum value at pH < 6.0. This shows that the FDH of *Methylacidiphilum* sp. RTK17.1 is less active at the pH values mirroring cytosolic acidification and therefore could be vulnerable to formic acid-induced pH stress. Although very little is known about the pH dependence of FDH in acidophiles, similar pH-dependent activities have been reported in several neutralophiles (Schauer and Ferry, 1982; Axley et al., 1990), anaerobes (Liu and Mortenson, 1984), and fungi (Altaş et al., 2017). In addition, the presence of several acid-labile SH groups have been attributed to the circumneutral pH optima (pH 6.5–7.5) displayed by the NAD^+^-dependent FDH of *Methylosinus trichosporium* OB3b (Trotsenko and Murrell, 2008). Collectively, these data indicate that a formic acid-induced pH stress, of sufficient magnitude, can “*overwhelm*” the catabolic machinery of *Methylacidiphilum* sp. RTK17.1, thus precipitating a nonrecoverable collapse of the proton motive force. The absence of growth on formic acid, in repeated *Methylacidiphilum* sp. RTK17.1 batch experiments, is further evidence of organic acid-induced cytosolic acidification and is consistent with reports that formic acid does not support growth in *M. infernorum* V4 (Dunfield et al., 2007), and inhibits growth in *Methylacidiphilum fumariolicum* SolV at acidic pH values (Pol et al., 2007).

### Stable Growth of *Methylacidiphilum* sp. RTK17.1 on Formic Acid Is Possible in Continuous Culture

**Figure 3:**
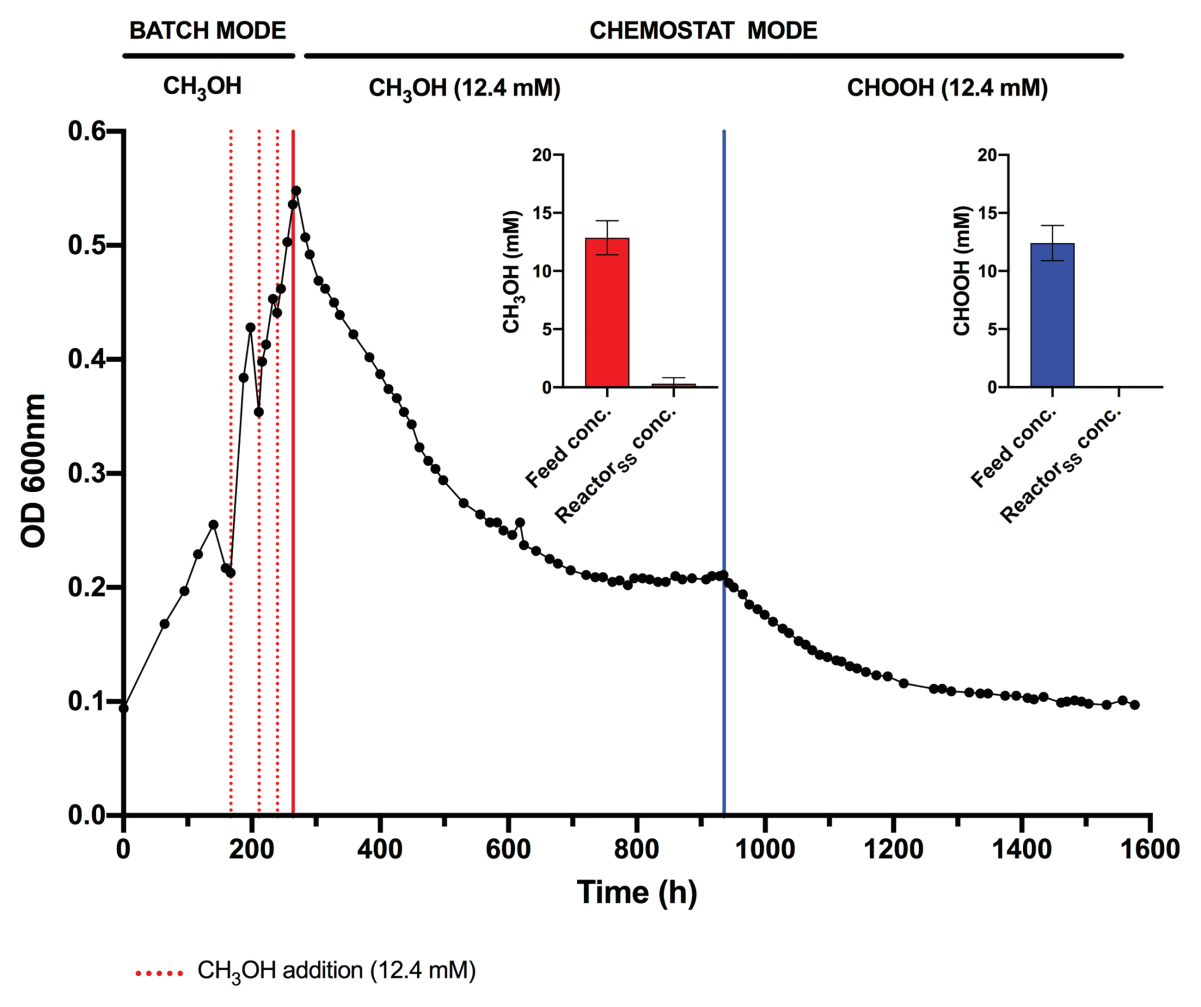
Chemostat cultivation of *Methylacidiphilum* sp. RTK17.1 on methanol (CH_3_OH) and formic acid (CHOOH). For all conditions, cells were grown on V4 medium at 45°C, pH 2.5 with an agitation of 450 rpm and continuous gas supply (2% O_2_, 2% CO_2_, balance N_2_ (v/v); 150 ml min^−1^). Dashed red lines denote the supplemental addition of methanol (12.4 mM) during batch growth. Solid red and blue lines indicate the transition to chemostat mode (D = 0.0052 h^−1^) on methanol and formic acid, respectively (supplied at 12.4 mM for both). Inset panels denote the influent feed concentration (feed conc.) and steady-state reactor concentration (Reactor_SS_ conc.) of methanol (red) and formic acid (blue) detected by high-performance liquid chromatography (HPLC) during chemostat operation. Error bars represent the standard deviation of triplicate measurements.

The lack of detectable growth on formate/formic acid by *Methylacidiphilum* sp. RTK17.1, although rationalized as a failure of pH homeostatic machinery, poses a metabolic quandary for this taxon. Formate clearly serves as an intermediate in the oxidation of methane to CO_2_ *via* an FDH-catalyzed reaction (Dunfield et al., 2007; Khadem et al., 2012c), yet conversely appears not to support growth as a sole substrate. Indeed, the oxidation of formate as a sole energy source should theoretically be capable of supporting CO_2_ fixation and growth in *Methylacidiphilum* spp. as it does with some neutralophilic methanotrophs (Kemp and Quayle, 1967; Bowman, 2014). To test this hypothesis, we attempted to grow *Methylacidiphilum* sp. RTK17.1 on formic acid in a steady-state continuous culture, the principle being that chemostat growth would minimize induced pH stress under formic acid-limiting growth conditions. To do this, we initially grew *Methylacidiphilum* sp. RTK17.1 on methanol in a chemostat before switching to formic acid in the nutrient feed. In contrast to comparable batch experiments at 12.4 mM formic acid, our findings show that *Methylacidiphilum* sp. RTK17.1 is capable of sustained (chemostat) growth using formic acid as the sole metabolizable source of energy (Figure 3). As evidenced by the absence of their detection in chemostat effluent streams (<0.15 mM), methanol and formic acid were the growth-limiting nutrients in their respective steady-state growth conditions (Figure 3). Biomass yields (as determined by cell dry weight; gCDW) were 7.83 (±0.41) gCDW mol^−1^ during growth on methanol and decreased by 63% to 2.86 (±0.27) gCDW mol^−1^ during growth on formic acid (Table 1). We did not assess the extent of intracellular glycogen produced as this has been previously reported in *Methylacidiphilum* spp. RTK17.1 (Carere et al., 2019) and SolV (Khadem et al., 2012b). It is noteworthy to mention that during both methanol‐ and formic acid-dependent chemostat growth, CO_2_ was continuously supplied in excess. While we cannot confirm that the oxidation of formic acid was able to supply both the necessary reducing equivalents (i.e., NADH) and inorganic carbon (CO_2_) required for cell growth, a previous report of methane-dependent growth in *Methylacidiphilum fumariolicum* SolV has shown that the oxidation of methane alone is able to sustain growth as long as evolved CO_2_ in the headspace reaches a minimum threshold concentration of 0.3% (v/v; Khadem et al., 2011). Biomass yields (g mol^−1^) on methanol and formic acid were ~115 and ~42%, respectively, of the values previously reported during growth of *Methylacidiphilum* sp. RTK17.1 on methane (Carere et al., 2017, 2019). However, on a per mole electron equivalent basis (Y_CDW/e_), biomass yields were greater for both methanol‐ and formic acid-grown cultures than for methane grown cells (Table 1). This likely reflects the additional energetic input necessary to catalyze the oxidation of methane to methanol *via* methane monooxygenase (CH_4_ + O_2_ + [NAD(P)H + H^+^]/QH_2_ → CH_3_OH + NAD(P)^+^/Q + H_2_O; Carere et al., 2017).

**Table 1:** Steady state biomass productivity of *Methylacidiphilum* sp. RTK17.1 during growth on methane (CH_4_), methanol (CH_3_OH), and formic acid (CHOOH)^∗^.

Biomass	CH_4_^a^	CH_4_^b^	CH_3_OH^c^	CHOOH^d^
Biomass productivity (mg L^−1^ h^−1^)	5.57 (±0.50)	5.40 (±0.26)	13.18 (±0.70)	4.73 (±0.45)
Y_CDW/substrate_ (g mol^−1^)	6.29 (±0.25)	6.79 (±0.55)	7.83 (±0.41)	2.86 (±0.27)
Y_CDW/e−_ (g mol^−1^)	0.78 (±0.03)	0.85 (±0.07)	1.31 (0.07)	1.43 (±0.14)

∗ The standard deviation of minimum triplicate measurements is provided in brackets.

a *Methylacidiphilum* sp. RTK17.1 grown continuously on V4 mineral medium (50°C, pH 2.5) at 10 ml min^−1^ 14.1% O_2_, 0.4% H_2_, 3.2% CH_4_, 26% CO_2_, with the balance N_2_ (v/v). A constant dilution rate (D = 0.02 h^−1^) was maintained, with NH_4_^+^ supplied at an influent concentration of 0.4 g L^−1^ (Carere et al., 2019).

b *Methylacidiphilum* sp. RTK17.1 grown continuously on V4 mineral media (50°C, pH 2.5) at 10 ml min^−1^ 3.5% O_2_, 3% CH_4_, 26% CO_2_, balance N_2_ (v/v). A dilution rate of 0.02 h^−1^ was maintained (Carere et al., 2017).

c *Methylacidiphilum* sp. RTK17.1 grown continuously on V4 mineral medium supplemented with 12.4 mM CH_3_OH, 45°C, pH 2.5, D = 0.0052 h^−1^ in a 2% CO_2_, 2% O_2_, balance N_2_ (v/v) headspace (This study).

d *Methylacidiphilum* sp. RTK17.1 grown continuously on V4 mineral medium supplemented with 12.4 mM CHOOH, 45°C, pH 2.5, D = 0.0052 h^−1^ in a 2% CO_2_, 2% O_2_, balance N_2_ (v/v) headspace (This study).

### Transcriptome Analysis Reveals Key Changes in the Expression of Genes Relating to Energy/Carbon Metabolism and pH Homeostasis

**Figure 4:**
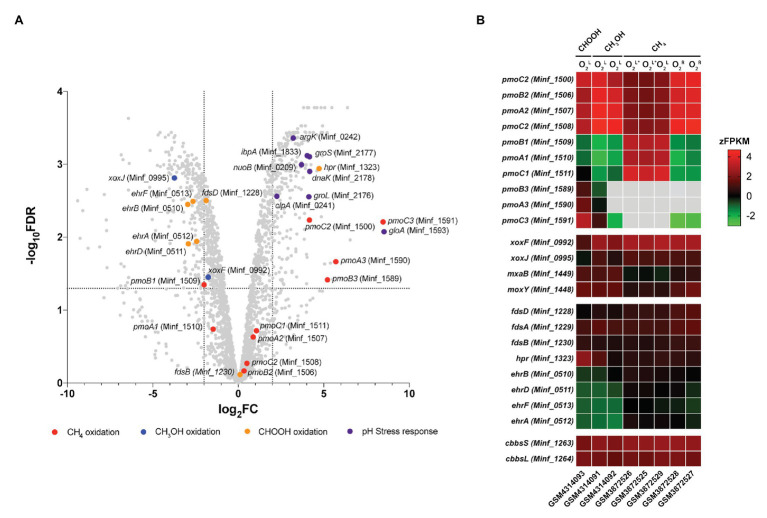
Differential gene expression profiles of chemostat-grown cultures of *Methylacidiphilum* sp. RTK17.1 grown on methanol (CH_3_OH) and formic acid (CHOOH). **(A)** Volcano plot showing differential gene expression changes following the transition from growth on methanol to growth on formic acid. Fold-change values (log_2_FC) and false discovery rates (FDR) are calculated using methylotrophic growth as the reference condition. Each gene is represented by a gray dot and genes of interest are highlighted as per the legend. Dashed horizontal and vertical lines signify FDR = 0.001 and log_2_FC = ∣2∣, respectively. **(B)** Heat map of transcript abundance for key genes encoding the structural subunits of enzymes participating in methane oxidation (*pmoBAC*; particulate methane monooxygenase), methanol oxidation (*xoxFJ*, *mxaB*, and *moxY*), formate oxidation (*fdsDAB*, *hpr*, and *ehrBDAF*), and carbon dioxide fixation (*cbbsSL*). Expression data is displayed as the z-normalization of fragment counts per kilobase million transcripts (zFPKM; Hart et al., 2013) for steady-state formic acid (CHOOH, GSM4314093), methanol (CH_3_OH, GSM4314092, GSM4314091), and methane (CH_4_, GSM3872525-GSM3872529) grown cultures under oxygen limited (O_2_^L^), oxygen, and ammonia limited (O_2_^L∗^) and oxygen replete (O_2_^R^) condition. Gray boxes denote expression values where zFPKM < −3.

Transcriptome analysis was performed on steady-state *Methylacidiphilum* sp. RTK17.1 cultures to determine whether genes associated with pH homeostasis, energy metabolism, and/or carbon metabolism were regulated in response to growth on formic acid (Figure 4). To account for the lack of replicates in the formic acid growth condition, complementary expression analyses were performed against methane-grown cultures described previously (Carere et al., 2019). In response to the transition from methanol to formic acid, 188 genes were identified as significantly upregulated, and 193 genes were significantly downregulated [Log_2_FC ≥ |2|, *p* < 0.001; false discovery rate (FDR) < 0.05].

The genomes of *Methylacidiphilum* spp. are known to encode up to three paralogous pMMO operons (*pmoCAB*; in addition to a fourth orphaned *pmoC*) that are phylogenetically divergent from one another by up to 50% amino acid identity (Op den Camp et al., 2009). This includes three *pmoCAB* operons in *Methylacidiphilum* spp. Kam1, SolV, V4, Phi, Fur, FdI, and RTK17.1, and only two complete copies of *pmoCAB* in strains Rib and Ice. Only one complete *pmoCAB* cluster has been identified in strain Yel, along with two truncated *pmoAB* and *pmoCA* clusters (Erikstad et al., 2019). Multiple pMMOs, with differing apparent K_m_ values, are also characteristic of the type II methanotrophs (Baani and Liesack, 2008), and it has been proposed that this multiplicity provides survival advantages in niches where substrate concentrations may be variable. Likewise, in *Methylacidiphilum* spp. Kam1, SolV and V4, it has been shown that each of the *pmoCAB* operons are highly conserved and under intense purifying selection, further suggesting that they have evolved to fulfill distinct physiological roles (Kruse et al., 2019). Accordingly, the differential regulation of the *pmoCAB1* and *pmoCAB2* operons in *Methylacidiphilum* sp. RTK17.1 (Carere et al., 2019) and *M. fumariolicum* SolV (Khadem et al., 2012a) has previously been demonstrated in response to oxygen availability. Recently, expression of the *pmoCAB3* operon was reported during the growth of *M. fumariolicum* SolV on methanol/ethane and methanol/propane (Picone et al., 2020). This led authors to suggest that *pmoCAB3* expression was linked to the absence of methane or the presence of methanol and to note that expression levels increased when propane was supplied.

In this study, growth of *Methylacidiphilum* sp. RTK17.1 on methanol was accompanied by high levels of *pmoCAB2* (Minf_1506–1508) expression. Considering that methylotrophic growth was oxygen limited, this result is consistent with our previous work (Carere et al., 2019). Congruently, the closely related, and proximally located, *pmoCAB1* operon (homologous to *M. infernorum* V4 loci Minf_1509–1511) that is highly expressed during oxygen-replete growth on methane was only weakly transcribed. As observed in *M. fumariolicum* SolV (Picone et al., 2020), methylotrophic growth of *Methylacidiphilum* sp. RTK17.1 was accompanied by very weak expression of the *pmoCAB3* operon (average FPKM = 331; Supplementary Table S1). Surprising, however, was the observation that formic acid-dependent growth strongly stimulated expression of the *pmoCAB3* operon. When growing on formic acid, subunits of the particulate methane monooxygenase operon (*pmoCAB3*), corresponding to *M. infernorum* V4 loci Minf_1589–1591, were highly expressed (average FPKM = 18,422) and displayed the greatest degree of transcriptional upregulation (average: 6.5 Log_2_FC, *p* < 0.009) observed (Figure 4B; Supplementary Table S1). Although the physiological role of the *pmoCAB3* operon remains unknown, these collective data suggest that it may encode a high-affinity methane monooxygenase that exhibits some promiscuity toward other alkanes. Given the challenges associated with the heterologous expression of pMMO (Chan et al., 2011), future biochemical and biophysical studies aiming to characterize this divergent *pmoCAB3* operon may benefit from using a similar chemostat cultivation-dependent approach to enrich for its expression.

Transcripts for each of the genes required for the complete oxidation of CH_4_, carbon fixation, and glycogen synthesis were detected (Supplementary Table S1), thereby indicating that *Methylacidiphilum* sp. RTK17.1 remains primed for methanotrophic growth. Nevertheless, in response to formic acid-dependent growth, genes encoding the lanthanide-dependent methanol dehydrogenase, *xoxF* (Minf_0992; −1.72 Log_2_FC, *p* = 0.014) and *xoxJ* (Minf_0995; −3.69 Log_2_FC, *p* < 0.001), were down regulated. Transcriptional regulation of these XoxF-type methanol dehydrogenase genes has previously been reported in response to lanthanide availability in the methanotroph *Methylorubrum extorquens* AM1 (Good et al., 2019). Conversely, the NAD^+^-dependent formate dehydrogenase encoded by Minf_1323 (*hpr*) was upregulated (4.72 Log_2_FC, *p* < 0.001) and highly transcribed (FPKM: 7408) in response to growth on formic acid. The other putative formate dehydrogenases, including a hetero-multimeric Mo-containing formate dehydrogenase (Minf_1228–1231; *fdsBAD*) and a formate hydrogen lyase (*ehrBDAF*) were only weakly transcribed under all growth conditions (Supplementary Table S1). The presence of multiple formate dehydrogenases within methanotroph genomes is not unusual (Chistoserdova et al., 2004, 2007; Crowther et al., 2008); however little is known about the physiological role these enzymes may play in acidophilic methanotrophs. Differential expression of FDH genes in response to molybdenum or tungsten availability, in the facultative methylotroph *Methylobacterium extorquens* AM1, suggests that the presence of multiple FDHs may provide a means for increased ecological fitness (Chistoserdova et al., 2004).

Transcriptome analysis also revealed that several genes commonly associated with bacterial stress response were differentially expressed during growth on formic acid (Figure 4A). *Methylacidiphilum* sp. RTK17.1 upregulated the expression of molecular chaperone heat-shock proteins *groES* (Minf_2177; 4.16 Log_2_FC, *p* < 0.001), *groEL* (Minf_2176; 4.12 Log_2_FC, *p* < 0.001), *dnaK* (Minf_2178; 4.17 Log_2_FC, *p* < 0.001), and *ipbA* (Minf_1833; 4.03 Log_2_FC, *p* < 0.001) during growth on formic acid (Figure 4B). These proteins encode key factors that prevent the misfolding and aggregation of ribosome-bound polypeptides and have shown upregulation in response to acid (Len et al., 2004), oxidative and heat stress (Kitagawa et al., 2002). Likewise, homologs to the stress response protein-arginine kinase (*mcsB;* Minf_0242) and *clpA* unfoldase (Minf_0241) of *Bacillus subtilis* (Elsholz et al., 2011) were upregulated 3.21‐ and 2.26-fold, respectively, (*p* < 0.001). Interestingly, the most highly upregulated gene in response to growth on formic acid (8.52 Log_2_FC, *p =* 0.002) putatively encodes a methylmalonyl-CoA epimerase (Minf_1593, *gloA*). This enzyme is involved in the metabolism of propionate, branched-chain amino acids, odd-chain fatty acids, and the reversible conversion of (S)-methylmalonyl-CoA to succinyl-CoA. Regulation of *gloA* has previously been implicated in acid stress response in *Propionibacterium acidipropionici* (Guan et al., 2014); however, its role in *Methylacidiphilum* sp. RTK17.1 is not yet clear. One possibility is that the *gloA* transcription is linked to the proximate *pmoCAB3* operon (Minf_1589-1591) that is strongly upregulated. Finally, although the use of transporters to catalyze active proton or cation transport is a common strategy for bacterial pH homeostasis (Slonczewski et al., 2009), transcriptional regulation is not always observed. In support of this, constitutive expression of genes encoding a K^+^ translocating ATPase (Minf_0033-0035; *kpdCBA*) was observed under all growth conditions. As observed in other acidophiles (Baker-Austin and Dopson, 2007), it seems likely *Methylacidiphilum* sp. RTK17.1 actively imports K^+^ (electrogenic uptake) as a strategy to generate its reversed membrane potential, thereby minimizing the inward flux of H^+^ and facilitating pH homeostasis.

Our findings show that the proton motive force (PMF) for this species is primarily generated by a pH gradient across the cellular membrane. In batch experiments, the addition of formic acid resulted in no observable cell growth and, correspondingly, acidification of the cytosol. Nevertheless, stable growth on formic acid as the sole source of metabolizable energy was demonstrated in continuous cultures following the transition from methanol-dependent growth. Under these conditions, biomass yields on formic acid were nearly equivalent on a per mole electron basis to methanol-grown cells. The transition to growth on formic acid, however, coincided with transcriptional upregulation of several genes associated with an acid-stress response. These results therefore highlight the advantages of using chemostats to complement batch-culture experiments for the physiological characterization of microbial species. This has been well demonstrated with *Methylacidiphilum* spp. where otherwise cryptic phenotypes, such as lithoautotrophic and mixotrophic growth on H_2_ (Mohammadi et al., 2016; Carere et al., 2017, 2019), and ammonia oxidation (Mohammadi et al., 2017) were identified *via* genome analysis but were not demonstrable in batch-culture experiments (Hou et al., 2008; Op den Camp et al., 2009; Khadem et al., 2012c). It is likely that further cryptic phenotypes can be discovered using chemostat-based setups, not only in acidophilic methanotrophic strains but also for other microbial strains (Tamburini and Mastromei, 2000). Finally, these results also show that low concentrations of formic acid represent a utilizable source of energy/carbon to the acidophilic methanotrophs that are commonly found within geothermal environments and adds to previous research (Mohammadi et al., 2016; Carere et al., 2017, 2019) showing that metabolic flexibility in aerobic methane-oxidizing bacteria (methanotrophs) likely enhances cell growth and survival in environments where methane resources are variable or limiting.

## Conclusion

Very little is known about the pH homeostatic mechanisms used by acidophiles to accommodate the organic acids present within acidic environments. In this study, we have shown that formic acid represents a utilizable source of energy/carbon to the thermoacidophilic methanotroph, *Methylacidiphilum* sp. RTK17 at pH 2.5. During sustained growth, in response to the influx of formic acid across the cell membrane, *Methylacidiphilum* sp. RTK17.1 alters expression of key genes relating to energy/carbon metabolism and bacterial stress response. Findings reported in this study expand the known metabolic flexibility of verrucomicrobial methanotrophs to include organic acids while also highlighting the potential advantages of chemostat-culture experiments to characterize the physiology of acidophilic species.

## Data Availability Statement

The datasets presented in this study can be found in online repositories. The names of the repository/repositories and accession number(s) can be found in the article/Supplementary Material.

## Author Contributions

CC and MS conceived the study. CC, KHa, MS, and GC contributed to the experimental design. CC, LC, and KHo conducted the bioreactor and wet lab experiments. CC and KW performed the transcriptome analysis. CC, KHa, and GC performed the bioenergetic analysis. CC, KW, and MS wrote the manuscript with input from KHo, KHa, and GC. All authors contributed to the article and approved the submitted version.

### Conflict of Interest

The authors declare that the research was conducted in the absence of any commercial or financial relationships that could be construed as a potential conflict of interest.
