# Supplementary material for: Growth on Formic Acid Is Dependent on Intracellular pH Homeostasis for the Thermoacidophilic Methanotroph Methylacidiphilum sp. RTK17.1
Source: Front Microbiol. 2021 Mar 24;12:651744. doi: 10.3389/fmicb.2021.651744 (PMC8024496; doi:10.3389/fmicb.2021.651744)
Supplement: Supplementary file 1 [file Data_Sheet_1.pdf]

Table S1. Expression profiles (FPKM) of key genes encoded in the Methylacidiphilum sp. RTK17.1 genome in response to changes to chemostat growth operation. Amino acid orthologs to Methylacidiphilum infernorum V4 are shown.

Gene	Predicted Function	Gene ID *	***M. infernorum V4* ortholog**			**Expression (FPKM)**							
			**Accesion**	**locus tag**	**% ID**	**GSM4314093** CHOOH, O2-lim, NH4+	**GSM4314091** CH3OH, O2-lim, NH4+	**GSM4314092** CH3OH, O2-lim, NH4+	**GSM3872526** CH4, O2-lim, N2	**GSM3872525** CH4, O2-lim, N2, H2	**GSM3872529** CH4, O2-lim, NH4+, H2	**GSM3872528** CH4, O2-excess, NH4+, H2	**GSM3872527** CH4, O2-excess, NH4+
**Methane oxidation to methanol**													
pmoC1	particulate methane monooxygenase, C subunit	KU509367	WP_012463847.1	Minf_1511	99	36	24	75	40741	31686	41798	21	17
pmoA1	particulate methane monooxygenase, A subunit	KU509368	ABX56601.1	Minf_1510	100	2	9	27	12426	10352	14656	12	23
pmoB1	particulate methane monooxygenase, B subunit		WP_012463845.1	Minf_1509	99	36	24	75	40741	31686	41798	21	17
pmoC2	particulate methane monooxygenase, C subunit	KU509373	WP_048810233.1	Minf_1500	99	148211	34484	9615	2932	2740	3653	41842	67469
pmoA2	particulate methane monooxygenase, A subunit	KU509371	WP_012463843.1	Minf_1507	99	40317	74590	45278	3579	2911	3963	40041	51651
pmoB2	particulate methane monooxygenase, B subunit	KU509372	WP_012463842.1	Minf_1506	99	19444	53597	29766	3460	2735	4018	30932	47815
pmoC2	particulate methane monooxygenase, C subunit	KU509370	WP_012463844.1	Minf_1508	99	45082	104274	69195	4834	3926	5232	71719	120797
pmoC3	particulate methane monooxygenase, C subunit	KU509318	WP_012463927.1	Minf_1591	99	53094	760	22	2	2	2	4	4
pmoA3	particulate methane monooxygenase, A subunit	KU509319	WP_012463926.1	Minf_1590	99	1657	163	3	0	1	1	1	2
pmoB3	particulate methane monooxygenase, B subunit	KU509320	WP_012463925.1	Minf_1589	97	514	71	1	0	0	0	0	1
**Methanol oxidation to formate**													
xoxF	methanol dehydrogenase, large subunit	KU509410	WP_012463329.1	Minf_0992	99	491	5445	3470	10198	8630	12691	8291	8865
xoxJ [#]	unknown function	Ga0079954	WP_012463332.1	Minf_0995	99	20	911	514	1278	979	1138	933	838
mxaB	transcriptional regulator	KU509376	WP_012463785.1	Minf_1449	100	558	931	1409	169	173	139	388	499
moxY	methanol utilization control sensor protein	KU509377	WP_012463784.1	Minf_1448	100	1487	1471	1763	433	571	462	1090	1962
mxaR	ATPase involved in methanol oxidation	KU509402	WP_012463503.1	Minf_1166	97	6	12	11	15	17	14	13	19
**Formate oxidation to CO_2**													
fdsG	formate dehydrogenase, gamma subunit	KU509398	WP_048810187.1		97								
fdsB	formate dehydrogenase, beta subunit	KU509399	WP_012463566.1	Minf_1230	100	167	419	443	451	385	493	638	621
fdsA	formate dehydrogenase, alpha subunit	KU509400	WP_048810186.1	Minf_1229	99	389	1246	1066	914	911	846	1220	1388
fdsD	formate dehydrogenase delta subunit	KU509401	WP_048810185.1	Minf_1228	94	40	410	402	649	677	635	960	1145
hpr	formate dehydrogenase	KU509384	WP_012463659.1	Minf_1323	99	7408	1099	400	481	473	643	579	413

Table S1. Expression profiles (FPKM) of key genes encoded in the Methylacidiphilum sp. RTK17.1 genome in response to changes to chemostat growth operation. Amino acid orthologs to Methylacidiphilum infernorum V4 are shown.

ehrB	putative formate-dependent oxidoreductase complex subunit	KU509439	WP_012462850.1	Minf_0510	99	8	111	252	437	368	316	269	187
ehrD	putative formate-dependent oxidoreductase complex subunit	KU509438	WP_012462851.1	Minf_0511	99	4	60	112	304	282	230	266	97
ehrF	putative formate-dependent oxidoreductase complex subunit	KU509436	WP_012462853.1	Minf_0513	99	3	50	70	212	191	158	143	77
ehrA	putative formate-dependent oxidoreductase complex subunit	KU509437	WP_012462852.1	Minf_0512	99	3	49	51	374	263	276	231	110
ehrE	putative formate-dependent oxidoreductase complex subunit	KU509449	WP_012462854.1		100								
ehrG	putative formate-dependent oxidoreductase complex subunit	KU509435	WP_048810433.1		97								
**Hydrogen metabolism**													
*hyaF**	hydrogenase-1 operon protein	KU509388	WP_012463654.1	Minf_1318	99	8	47	375	398	326	430	47	45
*hyaC**	oxygen-tolerant membrane-bound [NiFe] - hydrogenase, cytochrome *b* subunit	KU509387	WP_012463655.1	Minf_1319	99	0	35	353	336	229	283	31	40
*hyaB**	oxygen-tolerant membrane-bound [NiFe] hydrogenase, large subunit	KU509386	WP_012463656.1	Minf_1320	99	25	181	1049	1932	1485	1802	148	134
*hyaA**	oxygen-tolerant membrane-bound [NiFe] hydrogenase, small subunit	KU509385	WP_048810203.1	Minf_1321	99	2	293	1235	2343	1926	2178	176	145
hyhB	NADP-coupled cytosolic bidirectional hydrogenase, FeS subunit	KU509477	WP_012464721.1	Minf_2387	99	431	375	625	294	290	232	174	211
crp	cyclic nucleotide-binding domain-containing protein	KU509476	WP_012464722.1	Minf_2388	97	209	253	433	215	201	164	112	138
hyhG	NADP-coupled cytosolic bidirectional hydrogenase, diaphorase subunit NAD(P)H-flavin reductase	KU509475	WP_012464723.1	Minf_2389	98	62	215	340	176	150	154	93	96
hyhS	NADP-coupled cytosolic bidirectional hydrogenase, small subunit	KU509474	WP_012464724.1	Minf_2390	98	48	203	330	146	121	130	96	71
hyhL	NADP-coupled cytosolic bidirectional hydrogenase, large subunit	KU509473	WP_012464725.1	Minf_2391	98	16	99	204	107	81	95	80	71
hypB	hydrogenase maturase protein, Ni storage	KU509355	WP_012464135.1	Minf_1799	99	409	368	575	826	1007	807	255	289
hypC	hydrogenase maturase protein, Fe- $(CN^-)_2$ -CO insertion	KU509357	WP_012464132.1	Minf_1796	100	1923	1082	2219	1102	1048	979	883	1021
hypD	hydrogenase maturase protein, Fe- $(CN^-)_2$ -CO insertion	KU509358	WP_012464131.1	Minf_1795	99	71	258	372	332	311	308	332	255
hypE	hydrogenase maturase protein, CN^- ligand biosynthesis	KU509359	WP_012464130.1	Minf_1794	99	14	73	180	159	149	171	135	119
hypF	hydrogenase maturase protein, CN^- ligand biosynthesis	KU509356	WP_012464133.1	Minf_1797	99	7	33	43	72	66	77	41	35
**Copper homeostasis**													
cueR	copper resistance operon regulatory protein	KU509440	WP_012462789.1	Minf_0449	99	1055	172	222	126	146	121	166	115

Table S1. Expression profiles (FPKM) of key genes encoded in the Methylacidiphilum sp. RTK17.1 genome in response to changes to chemostat growth operation. Amino acid orthologs to Methylacidiphilum infernorum V4 are shown.

copA	Cu(I)-translocating P-type ATPase/ multicopper oxidase, copper-binding site	KU509375	gb|ABX56608.1|		99								
cueO	multicopper oxidase family protein	KU509374	WP_048810228.1	Minf_1469	99	0	0	0	0	0	0	0	0
cusA	copper efflux pump	KU509461	WP_012462437.1	Minf_0095	99	27	76	90	78	77	77	77	79
cusB[#]	copper efflux pump membrane fusion protein	Ga0079954	WP_012462436.1	Minf_0094	98	77	95	151	132	132	133	131	116
cusS	copper sensor histidine kinase	KU509433	WP_012462941.1	Minf_0601	99	40	43	77	48	51	47	64	47
cusR	DNA binding copper response regulator	KU509432	WP_048810437.1	Minf_0602	99	325	216	371	414	301	337	288	243
**Coenzyme PQQ synthesis**													
pqqB	coenzyme PQQ synthesis protein B	KU509397	WP_012463570.1	Minf_1234	99	439.1	2028.2	1681	2171.4	1911.7	1877.8	1190.9	1961.3
pqqC	coenzyme PQQ synthesis protein C	KU509396	WP_012463571.1	Minf_1235	99	78	521.1	790.4	1110.1	938	981.6	499.2	629.4
pqqD	coenzyme PQQ synthesis protein D	KU509395	WP_048810188.1	Minf_1236	98	8.2	197.2	346.7	683.5	586.9	552.1	370.1	321.4
pqqD	coenzyme PQQ synthesis protein D	KU509434	WP_012462864.1	Minf_0524	97				16	13	16	16	13
pqqE	coenzyme PQQ synthesis protein E	KU509394	WP_048810189.1	Minf_1237	99	1503	335.3	297.1	496.7	524.5	436.2	352.8	303.1
pqqA	Coenzyme PQQ synthesis protein A	KY820885	WP_012463569.1	Minf_1233	100	10974	6538.3	4642.3	4451.4	4011.8	6380.9	5363.9	8399
No homologs of *pqqG pqqF*													
**Glycogen synthesis**													
mdoG	glycans biosynthesis protein	KU509364	WP_012463952.1	Minf_1616	98	6.1	26.1	25.8	41.9	39.1	42.7	19.6	26.5
mdoG	glycans biosynthesis protein	KU509365	WP_012463951.1	Minf_1615	99	3.3	37.4	28.4	91.5	92.4	74.1	59.9	46.7
	glucan elongaton module (mdoH-like)	KU509366	WP_048810251.1	Minf_1612	99	8.4	24.1	24.1	40.5	42.2	42.3	28.1	39.8
GH57	glycosyl hydrolase family 57	KU509382	WP_012463665.1	Minf_1329	99	81.2	181.7	327.1	208.2	172.3	189.9	188	155.1
amyA	alpha amylase, catalytic domain	KU509383	WP_012463664.1	Minf_1328	98	1255	359.3	771.7	155.1	157.3	136.5	196.8	226.7
glgA	glycogen synthase	KU509360	WP_012464092.1	Minf_1756	99	5.2	61.3	58.9	53.5	50	56.3	55.3	51.1
glgA	glycogen synthase (ADP-glucose)	KU509479	WP_048810560.1	Minf_2355	98	18.9	148.2	121.3	155.7	149.2	170.5	156.8	88.9
glgB	1,4-alpha-glucan branching enzyme	KU509409	WP_012463357.1	Minf_1020	99	6.9	115.4	127.2	113.7	101.5	96.1	112.3	71.6
glgC	glucose-1-phosphate adenylyltransferase	KU509460	WP_048810004.1	Minf_0180	99	71.2	178.2	222.3	174.7	193.6	183.5	132.4	157.7
glgP	starch phosphorylase	KU509451	WP_012462597.1	Minf_0255	99	386.3	508.2	543.5	330.1	302.1	309.9	354.1	243.4
glgP	starch phosphorylase	KU509431	WP_012462995.1	Minf_0658	99	91.7	252.5	344.4	403.2	417.6	380.9	288.9	246.9
glgP	starch phosphorylase	KU509429	WP_012463128.1	Minf_0791	99	72.3	238	375.2	299.7	256.2	308.1	329.3	261.6
gdb1	glycogen debranching enzyme (alpha-1,6-glucosidase)	KU509417	WP_012463227.1	Minf_0890	99	42.5	181.7	131.3	173.6	144.3	163.6	173.9	129.4
rfaG	glycosyltransferase	KU509418	WP_012463226.1	Minf_0889	99	37.1	26.1	34.8	40.7	48.2	36.5	40.3	48.5
glcD	glycolate oxidase	KU509414	WP_048810142.1	Minf_0895	98	376.1	367.5	240	258.1	207.4	279	270.4	245.4
glcD	glycolate oxidase FAD binding subunit	KU509413	WP_012463233.1	Minf_0896	98	69.3	90.4	117.2	146.8	139.5	152	154	186.5
aceB	malate synthase	KU509415	WP_012463229.1	Minf_0892	98	138.5	219.6	351.6	179.6	169.3	160.7	158.1	137.9
aceA	isocitrate lyase	KU509416	WP_048810457.1		98								
manB	phosphoglucomutase	KU509328	WP_012464463.1	Minf_2127	99								
**Calvin-Benson Cycle**													
cbbS	ribulose 1,5-bisphosphate carboxylase, small subunit	KU509390	WP_012463599.1	Minf_1263	98	2735	4059	3405	5640	5887	6494	5443	4825
cbbL	ribulose 1,5-bisphosphate carboxylase, large subunit	KU509389	WP_012463600.1	Minf_1264	99	3052	2169	1810	2623	2302	3101	2878	2120
cbbX	probable Rubisco expression protein CbbX	KU509391	WP_012463598.1	Minf_1262	99	487	1974	2150	2265	2116	2174	2028	1962
pgk	phosphoglycerate kinase	KU509406	WP_048810158.1	Minf_1032	98	163	805	1195	699	672	804	727	883
gapA	glyceraldehyde-3-phosphate dehydrogenase	KU509405	WP_012463370.1	Minf_1033	99	842	2087	2805	876	769	1139	1176	1122

Table S1. Expression profiles (FPKM) of key genes encoded in the Methylacidiphilum sp. RTK17.1 genome in response to changes to chemostat growth operation. Amino acid orthologs to Methylacidiphilum infernorum V4 are shown.

fbaA	putative fructose-bisphosphate aldolase	KU509327	WP_048810542.1		98								
fbaA2	fructose-bisphosphate aldolase, class II	KU509448	WP_012462629.1	Minf_0287	100	550	1193	708	1025	1050	1199	995	741
fbp	fructose-1,6-bisphosphatase I	KU509361	WP_012464020.1	Minf_1684	99	315	342	217	399	386	487	445	337
glpX	fructose-1,6-bisphosphatase II	KU509381	WP_012463695.1	Minf_1359	100	851	1082	975	1127	1127	1028	869	1116
tktB	transketolase	KU509393	WP_012463596.1	Minf_1260	99	70	863	998	900	766	864	976	747
xfp	xylulose-5-phosphate/fructose-6-phosphate phosphoketolase	KU509412	WP_012463240.1	Minf_0903	99	545	487	430	262	253	249	266	212
tpiA	triosephosphate isomerase	KU509407	WP_012463368.1	Minf_1031	98	58	627	1002	511	518	563	570	659
rpiB	ribose 5-phosphate isomerase B	KU509459	WP_012462539.1	Minf_0197	99	565	965	1330	875	968	850	826	954
prkB	phosphoribulokinase	KU509392	WP_012463597.1	Minf_1261	100	46	867	1177	946	870	1022	1078	790
rpe	ribulose-phosphate 3-epimerase	KU509354	WP_012464174.1	Minf_1838	99	47	239	213	319	318	357	295	193
**TCA cycle**													
gltA	citrate synthase	KU509447	WP_012462674.1	Minf_0334	99	232	523	361	536	667	505	592	332
acnA	aconitate hydratase	KU509408	WP_012463364.1	Minf_1027	99	226	212	269	149	173	118	262	101
icd	isocitrate dehydrogenase	KU509411	WP_012463276.1	Minf_0939	99	303	594	668	458	608	389	613	523
sucA	2-oxoglutarate dehydrogenase, E1 component	KU509464	WP_048810403.1	Minf_0067	98	639	535	1071	663	861	399	908	480
sucB	2-oxoglutarate dehydrogenase, E2 component, dihydrolipoamide succinyltransferase	KU509463	WP_012462410.1	Minf_0068	98	397	674	1140	597	600	618	860	487
lpdA	pyruvate/2-oxoglutarate dehydrogenase, E3 component, dihydrolipoamide dehydrogenase	KU509462	WP_012462411.1	Minf_0069	99	56	398	716	320	288	287	397	228
lpdA	pyruvate/2-oxoglutarate dehydrogenase, E3 component, dihydrolipoamide dehydrogenase	KU509362	WP_012463971.1	Minf_1635	98	10	148	230	340	384	315	301	268
sucD	succinyl-CoA synthetase subunit alpha	KU509403	WP_012463383.1	Minf_1046	99	46	573	664	428	415	350	699	269
sucC	succinyl-CoA synthetase subunit beta	KU509404	WP_048810162.1	Minf_1045	99	34	322	455	222	372	162	582	161
sdhB	succinate dehydrogenase, catalytic subunit	KU509442	WP_012462728.1	Minf_0388	99	131	281	209	281	292	295	304	196
sdhA	succinate dehydrogenase, flavoprotein subunit	KU509441	WP_012462729.1	Minf_0389	98	3	25	20	19	29	31	31	33
sdhC	succinate dehydrogenase, cytochrome b subunit	KU509443	WP_012462727.1	Minf_0387	98	251	139	142	236	405	180	186	215
fumC	fumarase	KU509428	WP_012463130.1	Minf_0793	100	34	237	324	268	213	251	296	201
mdh	malate dehydrogenase	KU509380	WP_048810212.1		99								
**ATP Synthase**													
atpD	F-type H^+-transporting ATPase subunit beta	KU509426	WP_048810122.1	Minf_0839	99	357	193	169	185	152	172	143	135
atpC	F-type H^+-transporting ATPase subunit epsilon	KU509425	WP_012463177.1	Minf_0840	99	79	180	249	248	230	218	187	187
	ATP synthase protein I	KU509424	WP_012463178.1	Minf_0841	98	99	170	268	122	112	110	86	100
atpB	ATP synthase F0 subcomplex A subunit	KU509423	WP_012463179.1	Minf_0842	98	134	123	192	123	125	112	114	100
atpE	F-type H^+-transporting ATPase subunit c	KU509422	WP_012463180.1	Minf_0843	98	51	68	108	216	211	186	159	169
atpF	ATP synthase F0 subcomplex B subunit	KU509421	WP_012463181.1	Minf_0844	99	81	277	287	404	433	434	294	335

Table S1. Expression profiles (FPKM) of key genes encoded in the Methylacidiphilum sp. RTK17.1 genome in response to changes to chemostat growth operation. Amino acid orthologs to Methylacidiphilum infernorum V4 are shown.

atpA	F-type H+-transporting ATPase subunit alpha	KU509420	WP_012463182.1	Minf_0845	99	36	144	235	170	128	158	136	112
atpG	F-type H+-transporting ATPase subunit gamma	KU509419	WP_012463183.1	Minf_0846	100	27	161	295	174	148	133	127	124
atpH	F-type H^+-transporting ATPase subunit delta	KU509469	WP_012464754.1	Minf_2420	99	31	601	311	1223	955	1357	1184	686
atpB	F-type H^+-transporting ATPase subunit a	KU509472	WP_012464751.1	Minf_2417	99	78	1275	840	1476	1257	1803	1203	1133
atpE	F-type H^+-transporting ATPase subunit c	KU509471	WP_012464752.1	Minf_2418	100	310	2830	1818	4232	4176	4585	3688	3551
atpF	F-type H^+-transporting ATPase subunit b	KU509470	WP_048810391.1	Minf_2419	99	150	1894	873	3863	4047	4534	3468	2947
atpA	F-type H^+-transporting ATPase subunit alpha	KU509468	WP_012464755.1	Minf_2421	99	1628	1328	912	1982	1905	2072	2078	1304
atpG	F-type H^+-transporting ATPase subunit gamma	KU509467	WP_012464756.1	Minf_2422	99	152	566	416	1154	1322	1321	1402	979
atpD	F-type H^+-transporting ATPase subunit beta	KU509466	WP_012464757.1	Minf_2423	100	299	1034	1007	1672	1691	1741	1822	1301
atpC	F-type H^+-transporting ATPase subunit epsilon	KU509465	WP_012464758.1	Minf_2424	100	471	1705	1810	2665	3221	3128	2427	2401
**NADH dehydrogenase**													
nuoB	NADH-quinone oxidoreductase subunit B	KU509458	WP_012462551.1	Minf_0209	99	12895	3169	2268	1179	1036	989	1196	589
nuoA	NADH-quinone oxidoreductase subunit A	KU509480	WP_012464634.1	Minf_2300	99	360	923	897	559	686	555	499	654
nuoC	NADH-quinone oxidoreductase subunit C	KU509457	WP_012462552.1	Minf_0210	100	438	992	792	1278	1198	1451	1779	1059
nuoD	NADH-quinone oxidoreductase subunit D	KU509456	WP_012462553.1	Minf_0211	99	139	548	807	889	752	864	1003	586
nuoE	NADH-quinone oxidoreductase subunit E	KU509455	WP_012462554.1	Minf_0212	99	117	591	788	1168	1381	1091	1457	929
nuoF	NADH-quinone oxidoreductase subunit F	KU509454	WP_012462555.1	Minf_0213	99	68	421	582	874	972	921	1204	641
nuoG	NADH-quinone oxidoreductase subunit G	KU509453	WP_012462556.1	Minf_0214	99	131	895	1085	1190	1155	1062	1370	814
nuoH	NADH-quinone oxidoreductase subunit H	KU509452	WP_012462557.1	Minf_0215	99	16	127	148	345	417	360	393	319
nuoI	NADH-quinone oxidoreductase subunit I	KU509321	WP_048810348.1	Minf_2216	99	29	257	199	341	304	353	529	230
nuoJ	NADH-quinone oxidoreductase subunit J	KU509322	WP_048810347.1	Minf_2215	99	89	449	416	1100	1014	1179	1212	786
nuoK	NADH-quinone oxidoreductase subunit K	KU509323	WP_012464548.1	Minf_2214	100	149	379	324	784	593	706	724	441
nuoL	NADH dehydrogenase subunit L	KU509324	WP_012464547.1	Minf_2213	98	15	291	265	473	417	463	528	304
nuoM	NADH-quinone oxidoreductase subunit M	KU509325	WP_012464546.1	Minf_2212	99	5	107	69	280	306	338	412	237
nuoN	NADH dehydrogenase subunit N	KU509326	WP_012464545.1	Minf_2211	99	4	104	71	306	305	362	531	247
**Cytochrome C oxidase and Complex III (ACIII) (40)**													
cyoE	protoheme IX farnesyltransferase	KU509378	WP_012463763.1	Minf_1427	99	88	97	82	354	382	374	67	80
ctaA	cytochrome c oxidase assembly protein subunit 15	KU509379	WP_012463762.1	Minf_1426	99	1130	295	250	694	806	691	144	216
cyoC	cytochrome c oxidase subunit 3	KU509342	WP_012464286.1	Minf_1950	99	420	494	526	1544	1502	1579	724	576
cyoA	cytochrome c oxidase subunit 2	KU509344	WP_012464280.1	Minf_1944	100	2640	1092	1741	1731	1540	1484	1018	823
cyoB	cytochrome c oxidase subunit 1	KU509341	WP_012464287.1	Minf_1951	99	1297	559	405	1103	1132	1153	946	684
	caa$_3$-type oxidase, subunit IV	KU509343	WP_048810305.1	Minf_1949	98	183	294	367	1379	1698	1466	678	587
cccA	cytochrome c oxidase, *cbb*$_3$-type, subunit III	KU509340	WP_012464288.1	Minf_1952	100	44	212	184	391	402	432	420	323
cytC	cytochrome c	KU509481	WP_012463333.1	Minf_0996	97	13	929	548	1054	923	1085	1048	766
ccoO	cytochrome c oxidase *cbb*$_3$-type subunit 2	KU509339	WP_012464289.1	Minf_1953	99	19	170	152	262	201	235	253	149
ccoN	cytochrome c oxidase *cbb*$_3$-type subunit 1	KU509338	WP_048810306.1	Minf_1954	99	17	92	98	167	172	170	168	148
	cytochrome c & quinol oxidase polypeptide I	KU509482	WP_012462607.1	Minf_0265	91	20	130	249	4459	3684	4137	37	49

Table S1. Expression profiles (FPKM) of key genes encoded in the Methylacidiphilum sp. RTK17.1 genome in response to changes to chemostat growth operation. Amino acid orthologs to Methylacidiphilum infernorum V4 are shown.

	heme/copper-type cytochrome/quinol oxidase, subunit 1	KU509483	WP_012462607.1	Minf_0265	99								
ActG[†]	Hypothetical membrane-associated protein	Ga0079954	WP_012464292.1	Minf_1956	99	179	678	587	811	1131	1059	849	901
ActF[†]	quinol:cytochrome c oxidoreductase quinone-binding subunit 2	KU509337	WP_012464293.1	Minf_1957	99	35	103	86	152	170	171	189	146
cccA – ActE[†]	cytochrome c oxidase, cbb_3-type, subunit III	KU509336	WP_012464294.1	Minf_1958	99	47	236	371	195	182	221	195	153
ActD[†]	Hypothetical membrane-associated protein	Ga0079954	WP_012464295.1	Minf_1959	100	20	146	170	235	259	281	262	179
ActC[†]	prokaryotic molybdopterin-containing oxidoreductase family, membrane subunit	KU509335	WP_012464296.1	Minf_1960	99	29	121	97	142	132	137	178	104
hybA – ActB[†]	prokaryotic molybdopterin-containing oxidoreductase family, iron-sulfur binding subunit	KU509334	WP_012464297.1	Minf_1961	99	61	225	192	286	278	292	383	201
ActA[†]	cytochrome c7	KU509333	WP_012464298.1	Minf_1962	99	33	164	219	348	396	383	501	307
**Nitrogen fixation**													
nifH	Mo-nitrogenase iron protein subunit NifH	KU509347	WP_012464212.1	Minf_1876	99	69	22	24	9950	8773	28	30	52
nifV	homocitrate synthase	KU509346	WP_012464213.1	Minf_1877	99	7	5	0	4939	3460	11	12	49
nifD	Mo-nitrogenase MoFe protein subunit NifD precursor	KU509348	WP_012464210.1	Minf_1874	99	14	11	12	5600	5242	22	24	28
nifK	Mo-nitrogenase MoFe protein subunit NifK	KU509349	WP_048810527.1	Minf_1873	99	11	15	13	3696	3778	26	30	34
nifE	nitrogenase molybdenum-cofactor synthesis protein	KU509350	WP_012464208.1	Minf_1872	99	10	26	25	1743	1650	35	40	38
nifN	nitrogenase molybdenum-iron protein NifN	KU509351	WP_048810526.1	Minf_1871	99	4	21	17	1027	941	29	50	23
nifX	nitrogen fixation protein NifX	KU509352	WP_012464206.1	Minf_1870	100	2	16	13	1490	1423	35	76	32
**K+ transporting ATPase**													
kpdA	K+ transporting ATPase, Chain A		YP_001938694	Minf_0035		13.1	116.9	83.2	152.9	141.7	137.5	101.9	189.6
kpdB	K+ transporting ATPase, Chain B		YP_001938693	Minf_0034		4.4	91.4	98.3	137.5	139.9	136.1	108.5	199.8
kpdC	K+ transporting ATPase, Chain C		YP_001938692	Minf_0033		10.5	116	114.2	119.9	129.5	117	128	169.9
**Stress response**													
ibpA	molecular chaperone Hsp20	Ga0079954_121571	WP_012464169.1	Minf_1833		109106	16046	21106	10376	11652	13649	7098	10010
nuoB	NADH-quinone oxidoreductase subunit B	Ga0079954_1295	WP_012462551.1	Minf_0209	99	12895	3169	2268	1179	1036	989	1196	589
clpA	ATP-dependent Clp protease ATP-binding protein	Ga0079954_11272	WP_012462583.1	Minf_0241	99	4793	2242	3361	2497	2676	2371	2343	2311
argK	protein arginine kinase	Ga0079954_12126	WP_012462584.1	Minf_0242	100	6351	1832	1961	1522	1474	1337	1271	1051
groL	molecular chaperone GroEL	Ga0079954_121857	WP_012464512.1	Minf_2176	99	20587	4540	1783	7734	7666	9101	10824	5776
groS	molecular chaperone GroES	Ga0079954_121858	WP_012464513.1	Minf_2177	99	21738	3915	2705	10216	16106	15274	16788	11834
dnaK	chaperone protein DnaK	Ga0079954_121859	WP_012464514.1	Minf_2178	99	8162	1540	911	3352	4180	5176	6612	3458
gloA	glyoxalase	Ga0079954_121574	WP_012463929.1	Minf_1593	99	21567	297	9	0	0	1	1	1
